# Structural Relationship between Cerebral Gray and White Matter Alterations in Degenerative Cervical Myelopathy

**DOI:** 10.3390/tomography9010025

**Published:** 2023-01-31

**Authors:** Chencai Wang, Francesco Sanvito, Talia C. Oughourlian, Sabah Islam, Noriko Salamon, Langston T. Holly, Benjamin M. Ellingson

**Affiliations:** 1 UCLA Brain Tumor Imaging Laboratory (BTIL), Center for Computer Vision and Imaging Biomarkers, David Geffen School of Medicine, University of California Los Angeles, Los Angeles, CA 90024, USA; 2 Department of Radiological Sciences, David Geffen School of Medicine, University of California Los Angeles, Los Angeles, CA 90024, USA; 3 Unit of Radiology, Department of Clinical, Surgical, Diagnostic, and Pediatric Sciences, University of Pavia, 27100 Pavia, Italy; 4 Neuroscience Interdepartmental Graduate Program, David Geffen School of Medicine, University of California Los Angeles, Los Angeles, CA 90024, USA; 5 Department of Neurosurgery, David Geffen School of Medicine, University of California Los Angeles, Los Angeles, CA 90024, USA; 6 Department of Psychiatry and Biobehavioral Sciences, David Geffen School of Medicine, University of California Los Angeles, Los Angeles, CA 90024, USA

**Keywords:** degenerative cervical myelopathy, neurite orientation dispersion and density imaging, gray matter, white matter, structural relationship, mJOA

## Abstract

Patients with degenerative cervical myelopathy (DCM) undergo adaptive supraspinal changes. However, it remains unknown how subcortical white matter changes reflect the gray matter loss. The current study investigated the interrelationship between gray matter and subcortical white matter alterations in DCM patients. Cortical thickness of gray matter, as well as the intra-cellular volume fraction (ICVF) of subcortical whiter matter, were assessed in a cohort of 44 patients and 17 healthy controls (HCs). The results demonstrated that cortical thinning of sensorimotor and pain related regions is associated with more severe DCM symptoms. ICVF values of subcortical white matter underlying the identified regions were significantly lower in study patients than in HCs. The left precentral gyrus (*r* = 0.5715, *p* < 0.0001), the left supramarginal gyrus (*r* = 0.3847, *p* = 0.0099), the left postcentral gyrus (*r* = 0.5195, *p* = 0.0003), the right superior frontal gyrus (*r* = 0.3266, *p* = 0.0305), and the right caudal (*r* = 0.4749, *p* = 0.0011) and rostral anterior cingulate (*r* = 0.3927, *p* = 0.0084) demonstrated positive correlations between ICVF and cortical thickness in study patients, but no significant correlations between ICVF and cortical thickness were observed in HCs. Results from the current study suggest that DCM may cause widespread gray matter alterations and underlying subcortical neurite loss, which may serve as potential imaging biomarkers reflecting the pathology of DCM.

## 1. Introduction

Degenerative cervical myelopathy (DCM) is a potentially disabling sequalae of advanced cervical spondylosis, in which chronic spinal cord compression and injury can result in progressive neurological decline. Cervical stenosis occurs in the natural process of aging, affecting more than 5% of the population over the age of 40 [1].

Recent studies have demonstrated that DCM can induce cortical gray matter atrophy and microstructural white matter alterations of the brain [2,3,4]. Additionally, morphology- and microstructural connectivity-based analyses have been used to illustrate how cortical modification and reorganization of the sensorimotor system helps to compensate for functional loss caused by cervical spine degeneration [3,5,6,7]. For instance, the growth and proliferation of fiber pathways associated with sensorimotor functions, including the corona radiata and the posterior thalamic radiation, are more strongly correlated with increased quantitative anisotropy in DCM patients compared to healthy controls (HCs) [8]. However, it remains unknown whether underlying microstructural changes in the white matter reflect gray matter alterations.

One possible way to investigate the structural relationship between gray and white matter alterations in DCM patients is by characterizing the neurite density and the neurite orientation dispersion of the white matter underlying the thinning cortical regions (subcortical white matter). Using diffusion MRI, a previous study proposed the intracellular volume fraction (ICVF) as a voxel-wise estimation of the neurite density [9]. This refined microstructural metric was derived from neurite orientation dispersion and density imaging (NODDI), a biophysical model that assumes a priori tissue compartments in order to quantify the intra-neurite, extra-neurite, and free water compartments [9]. As NODDI-derived metrics have proven insightful for other neurological conditions [10,11], DCM studies may also benefit from their application.

Given the widespread changes in cerebral structure that have been previously reported, the current study aimed to explore the relationship between subcortical white matter alterations, as measured by variations in ICVF, and gray matter alterations, as measured by variations in cortical thickness, in DCM patients, as well as exploring their association with functional impairment. There is an emerging belief that the DCM disease spectrum actually begins with asymptomatic spinal cord compression (ASCC) and eventually deteriorates into symptomatic DCM in some patients [12,13,14,15]. Additionally, we have previously demonstrated that ASCC patients manifest brain changes that are different from those of asymptomatic healthy controls without spinal cord compression [16]. Therefore, we included ASCC patients in the current study in order to represent the entire spectrum of DCM pathogenesis, since understanding why some ASCC patients develop DCM and others do not remains a major gap in our knowledge base. We hypothesized that the microstructural alterations in subcortical white matter are reflective of gray matter thickness. Specifically, we theorized that (1) cortical thinning of sensorimotor and pain related regions is associated with more severe DCM symptoms; (2) the neurite density (measured by ICVF) of subcortical white matter underlying identified regions is lower in DCM and ASCC patients than in HCs; and (3) ICVF values are positively correlated with cortical thickness of gray matter in DCM and ASCC patients but not in HCs.

## 2. Materials and Methods

### 2.1. Patient Population

A total of 44 patients, including 37 DCM patients and 7 ASCC patients, were prospectively enrolled between 2016 and 2020 in a cross-sectional study involving observational MRI and evaluation of neck pain or neurological impairment. Each of the patients, recruited from an outpatient neurosurgery clinic, had spinal cord compression with deformation of the spinal cord and the absence of any visible cerebrospinal fluid signal around the spinal cord at the site of maximal compression. Patients presented with varying degrees of neck pain, neurological symptomatology, or both. All patients signed Institutional Review Board (IRB#11-001876; Medical IRB Committee #3; University of California Los Angeles) approved consent forms. All studies were conducted in compliance with the Health Insurance Portability and Accountability Act (HIPAA), and the UCLA IRB approved all aspects of the current study.

The entire study patient cohort (DCM and ASCC) comprised 27 males and 17 females, whose ages ranged from 37 to 81 years, with an average age of 59.9 years. Neurological or functional impairment was measured using the modified Japanese Orthopedic Association (mJOA) score [17], in which a lower value represents poorer neurological function. Additionally, a cohort of 17 neurologically intact, healthy controls (HCs) ranging in age from 25 to 62 years, with an average age of 41 years, underwent the same MRI protocols as the patient cohort. The difference in age between the two groups was significant (*t*-test, *p* < 0.001). The patient cohort and HCs demographic data are summarized in Table 1.

### 2.2. Presenting Symptoms

The most common presenting symptom was neck pain, which was experienced by 33 patients. Paresthesia in the upper extremities was reported by 30 patients. Gait dysfunction was found in 21 patients. Twenty patients presented with deterioration of hand function, commonly manifested by significant changes in their ability to perform certain daily activities, such as using utensils, writing, sewing, and buttoning buttons. Four patients had a history of bladder incontinence.

### 2.3. Physical Examination

Thirteen patients were noted to have weakness in the upper extremities on examination, and two had weakness in the lower extremities. Nine patients had decreased sensation in the upper extremities, and four had sensory changes in the lower extremities. Hyperreflexia was the most common sign of upper motor neuron dysfunction and was observed in 16 patients. Hoffman’s sign was observed in 14 patients. Four patients had clonus in the lower extremities, and three had a positive Babinski reflex.

### 2.4. Radiographical Imaging

Standard MRI was obtained in all patients and spinal cord compression was evident in each patient. Spinal canal narrowing was related to advanced cervical spondylosis manifested by a combination of facet arthropathy, ligamentum flavum hypertrophy, and varying degrees of ventral disc-osteophyte compression. Twenty-eight of the patients had a lordotic cervical spine, ten had straight, and seven had a kyphotic spinal alignment. Twenty-nine patients had T2-weighted signal abnormalities located within the spinal cord parenchyma and fourteen were without signal changes. Three patients had ossification of the posterior longitudinal ligament.

### 2.5. Diffusion Spectral Imaging (DSI) and T1-Weighted Structural MRI Acquisition

All MR images were collected using a Siemens Prisma 3T MR scanner (Siemens Healthcare, Erlangen, Germany). High-resolution 3-dimensional (3D) T1-weighted structural images were acquired using a magnetization-prepared rapid gradient-echo (MPRAGE) sequence in either the coronal, sagittal, or axial orientation. There was a repetition time (TR) of 2.3–2.5 s, a minimum echo time (TE), an inversion time (TI) of 900–945 ms, flip angle of 8–15°, field of view (FOV) 240 × 320 mm^2^, and matching matrix size of 240 × 320 for 1 mm^3^ isotropic voxel dimensions. In addition, diffusion spectral imaging (DSI) data were collected with a TR = 6.9–12.5 s; TE = 93 ms; flip angle of 90°, FOV of 245 × 245 mm^2^, with an acquisition matrix of 160 × 160 for a voxel size of 1.5 × 1.5 × 2.5 mm^3^. Following an acquisition without diffusion-sensitization (*b* = 0 s/mm^2^), 60 multi-shell diffusion-weighted images (quarter sphere sampling of *q*-space) were acquired with *b*-values of 250, 450, 650, 900, 1100, 1350, 1750, 1800, and 2000 s/mm^2^ in 3,6,4,3,12,12,2,4,15 directions, respectively.

### 2.6. T1-Weighted Structural Image Processing

**Figure 1:**
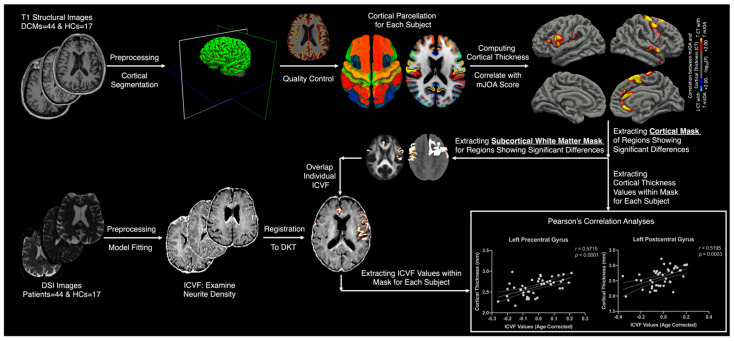
Image processing and analysis pipeline. Followed by the preprocessing of T1-weighted structural images, regions showing significant association between the cortical thickness and mJOA scores were identified. Average cortical thickness of those regions and subcortical white matter masks below those regions were extracted. Followed by the preprocessing of diffusion-weighted images, ICVF brain maps of each subject were extracted. Subcortical white matter masks of ROIs were superimposed on ICVF brain maps to extract regional ICVF values, which were used in further statistical analyses.

FreeSurfer (version 7.1; https://surfer.nmr.mgh.harvard.edu/fswiki; accessed on 19 December 2022) [18,19,20,21] was used to process T1-weighted structural images. Following the standard Freesurfer processing pipeline using the recon-all command, quality control was performed to verify accurate cortical segmentations. A full-width half-maximum (FWHM) of 10 mm was used to smooth the processed cortical surfaces, which were then registered to the FreeSurfer Desikan-Killiany (DKT) [22] standard space (Figure 1).

### 2.7. DSI Processing

All diffusion-weighted images were first de-noised using the *MRtrix3* package (http://www.mrtrix.org; accessed on 19 December 2022) [23], and then FMRIB’s Diffusion Toolbox (FDT) (www.fmrib.ox.ac.uk/fsl; accessed on 19 December 2022) was used for eddy current and patient motion artifact during image acquisition. Following skull extraction using Brain Extraction Tool (BET), the intracellular volume fraction (ICVF), which indicates the fraction of tissue water restricted within neurites (axons and dendrites) in the non-CSF compartment, was computed with the Accelerated Microstructure Imaging via Convex Optimization (AMICO) diffusion toolkit (https://github.com/daducci/AMICO; Daducci et al., 2015; accessed on 19 December 2022), using the neurite orientation dispersion and density imaging (NODDI) model. ICVF brain maps of each individual subject were registered to the FreeSurfer Desikan-Killiany (DKT) [22] standard space (Figure 1).

### 2.8. Whole-Brain Gray Matter Thickness Statistical Analysis

Following image preprocessing, a general linear model (GLM) was implemented through Freesurfer QDEC (Query, Design, Estimate, Contrast) to identify the thinning cortical regions associated with DCM. We correlated the cortical thickness with mJOA scores across the combined group of HC subjects and patients. Age, which has a predominantly linear relation to cortical thickness [21], was included as a covariate in this correlation analysis. The vertex-wise level of significance was set at *p* < 0.05, with multiple comparison corrections performed using Monte Carlo permutations with a significance level of *p* < 0.05. Identified regions, which reflect the significant areas of the cortical thickness associated with mJOA scores, were then used as regions of interests (ROIs) for further Pearson’s correlation analyses (Figure 1).

### 2.9. Region of Interest Subcortical White Matter Analyses

To measure the regional ICVF values for each subject and correlate them with cortical thickness, gray matter surface labels and masks were extracted from the ROIs showing significant association between cortical thickness and mJOA scores. Next, the subcortical white matter masks of the ROIs were created by sampling 1 mm into the white matter below the identified regions, starting from the gray and white matter boundary, in a direction perpendicular to the white matter surface. The subcortical white matter masks were then superimposed on ICVF maps of each subject. Extracted subcortical white matter ICVF values of each ROI were used for two analyses: (1) group-level differences in ICVF values (unpaired *t*-test) between patient and HCs cohorts, and (2) group-level Pearson’s correlation analysis between ICVF values and the cortical thickness across patient and HC cohorts, respectively. All analyses included age as a covariate with the level of significance set at *p* < 0.05 (Figure 1).

## 3. Results

### 3.1. Clinical Summary

The overall mean mJOA score for the study patient cohort (ASCC and DCM) was 15.3 (ranging from 9 to 18). The mJOA score for each of the ASCC patients was 18. The mean mJOA score for the 37 DCM patients was 14.7 (ranging from 9 to 17). Of the DCM patients, 25 had mild degenerative cervical myelopathy, 7 had moderate degenerative cervical myelopathy, and 5 had severe degenerative cervical myelopathy.

### 3.2. Structural Alterations

**Figure 2:**
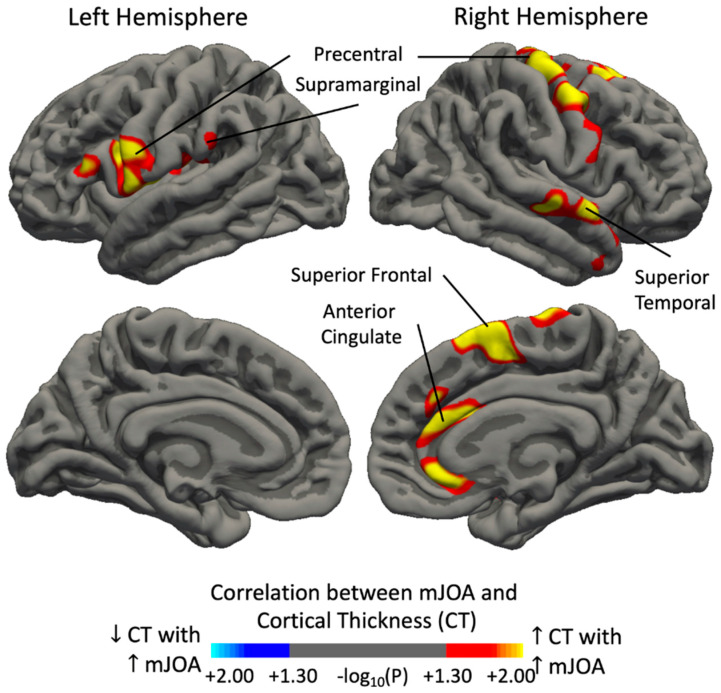
Regions demonstrating a strong association between the cortical thickness and mJOA score in a cohort of both patients and HCs. Red–yellow color denotes increasing cortical thickness/volume with better neurological status (increasing mJOA score), while blue–light blue color denotes decreasing cortical thickness/volume with better neurological status (increasing mJOA score). Significant clusters were determined by thresholding based on the level of statistical significance (*p* < 0.05).

When considering both study patients and HCs, cortical thickness was positively associated with mJOA in multiple regions (Figure 2), including the bilateral precentral gyri, right rostral and caudal anterior cingulate cortex, right superior frontal gyrus, right insular cortex, right superior temporal gyrus, and right caudal middle frontal gyrus, as well as the left supramarginal gyrus, left postcentral gyrus, left pars opercularis, and left pars triangularis. The ICVF values of subcortical white matter underlying all identified ROIs were found to be higher in HCs than in study patients (Table 2).

### 3.3. Region-Specific Structural Relationship

**Figure 3:**
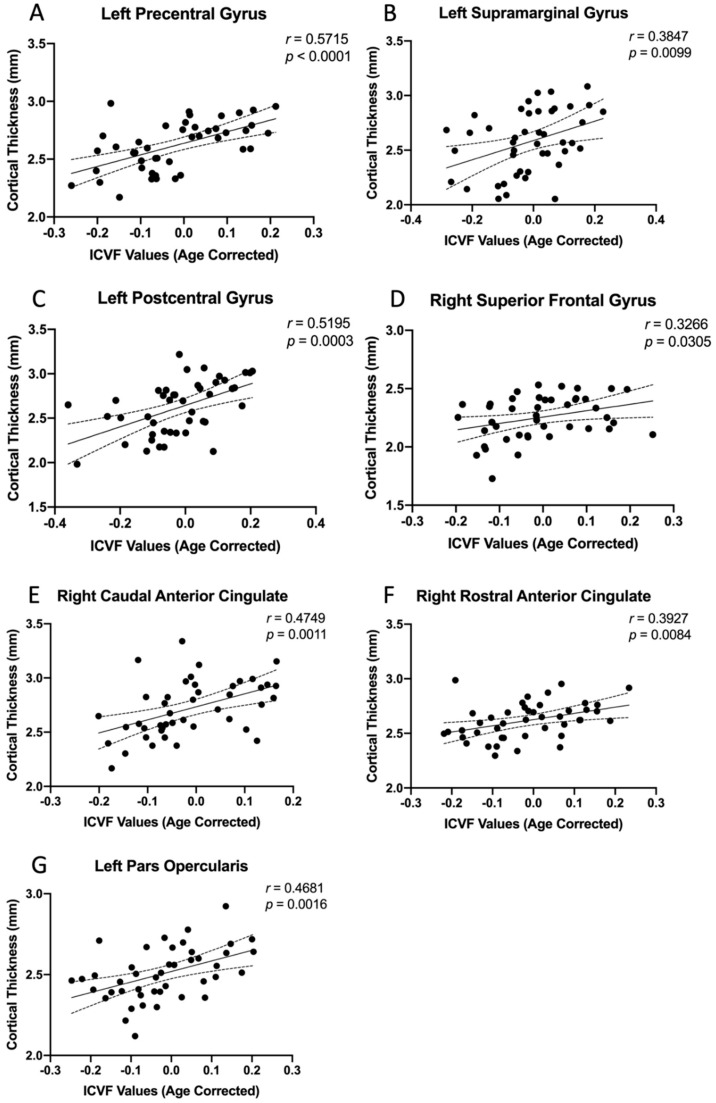
(**A**) The left precentral gyrus (*r* = 0.5715, *p* < 0.0001), (**B**) the left supramarginal gyrus (*r* = 0.3847, *p* = 0.0099), (**C**) the left postcentral gyrus (*r* = 0.5195, *p* = 0.0003), (**D**) the right superior frontal gyrus (*r* = 0.3266, *p* = 0.0305), (**E**) the right caudal anterior cingulate (*r* = 0.4749, *p* = 0.0011), (**F**) the right rostral anterior cingulate (*r* = 0.3927, *p* = 0.0084), and (**G**) the left pars opercularis (*r* = 0.4681, *p* = 0.0016), showing significant correlations between ICVF values and cortical thickness across 44 subjects in the study patient cohort.

To further test whether the microstructural changes were reflective of gray matter macrostructural characteristics, we performed group-level Pearson’s correlation analyses across subjects in the study patient and HC cohorts, respectively, within the cortical regions showing positive associations between the cortical thickness and mJOA score in Figure 2. When examining the association between the cortical thickness and the underlying subcortical white matter ICVF values of the same regions (Table 3), no significant relationship was observed across the HC cohort. On the contrary, the left precentral gyrus (Figure 3A, *r* = 0.5715, *p* < 0.0001), the left supramarginal gyrus (Figure 3B, *r* = 0.3847, *p* = 0.0099), the left postcentral gyrus (Figure 3C, *r* = 0.5195, *p* = 0.0003),the right superior frontal gyrus (Figure 3D, *r* = 0.3266, *p* = 0.0305), the right caudal anterior cingulate (Figure 3E, *r* = 0.4749, *p* = 0.0011), the right rostral anterior cingulate (Figure 3F, *r* = 0.3927, *p* = 0.0084), and the left pars opercularis (Figure 3G, *r* = 0.4681, *p* = 0.0016) demonstrated significant positive correlations between cortical thickness and the underlying subcortical white matter ICVF values in study patients.

## 4. Discussion

In the current study we examined the alterations of cortical thickness in DCM patients and ASCC patients. We demonstrated that the extent of these changes was correlated with degree of neurological impairment. Multiple imaging biomarkers have recently been proposed to identify cortical atrophy in regions responsible for the perception and integration of sensory information, motor regulation, and pain modulation [3,4,5,24]. Results from the current study were not only consistent with previous findings, but also provided seeding regions to investigate the structural relationships between gray and white matter alterations.

### 4.1. Gray and White Matter Alterations Associated with DCM

**Figure 4:**
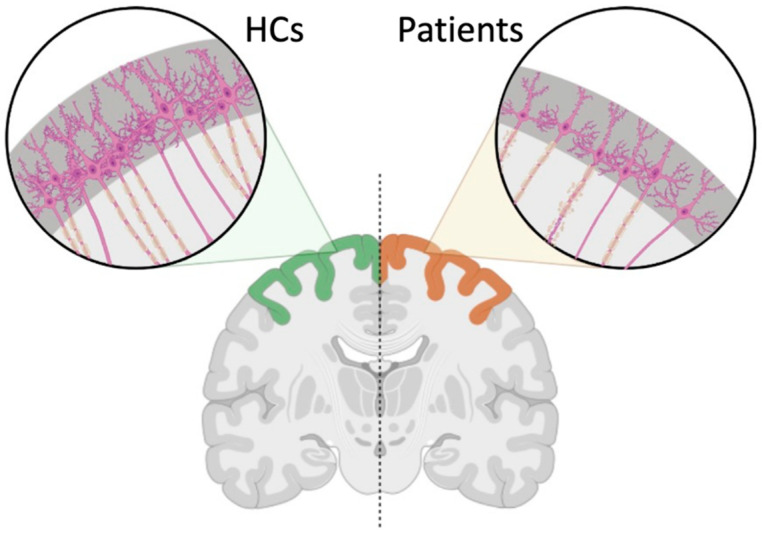
Interpretation of relationship between subcortical neurite density (measured by ICVF values) and cortical thickness in DCM patients compared to HC cohort.

NODDI-derived ICVF has been used to quantify the neurite density in brain tissue and describe the biological microstructures of axons and dendrites [9]. Previous studies have proposed that ICVF can provide information regarding subcortical white matter microstructure beyond DTI metrics and serve as possible markers for demyelinating disorders [10,11]. Although ICVF alterations and its associations with cortical thickness have not been reported in DCM patients, studies on multiple sclerosis and spinal cord injury discovered that primary pathology processes, such as demyelination or injury of the corticospinal tract, were often followed by sensorimotor cortical thinning [25,26]. Consistent with previous findings, we observed that the ICVF values of subcortical white matter underlying the altered gray matter regions were lower in the study cohort than in HCs. Additionally, the left precentral gyrus, the left supramarginal gyrus, the left postcentral gyrus, the right superior frontal gyrus, and the right anterior cingulate were thinner in study patients compared to HCs and demonstrated robust positive relationships between ICVF values and cortical thickness. These relationships were only observed in the study cohort and not in HCs, which further supported our hypothesis that the microstructural alterations associated with DCM are reflective of cortical thinning in sensorimotor and pain related regions. Taken together, cortical thinning and the consequential depletion of the neurite compartment in sensorimotor and pain related regions may suggest possible neural loss related to DCM (Figure 4). In addition, the fact that neurite compartment depletion in the subcortical white matter was observed beyond regions connected to the corticospinal tract, primary nociceptive, and sensory pathways suggests that DCM may cause a widespread subcortical neurite loss involving regions within cortex-to-cortex or basal ganglia-to-cortex connections.

It is worth noting that the current study observed neurite density loss in the right precentral gyrus, but cortical thickness of the right precentral gyrus was not significantly correlated with underlying white matter ICVF values. A study of Alzheimer’s disease reported that, while cortical neurite loss may be an early feature of the disease, as the disease advances the inherent pathophysiological relationship between ICVF and cortical thickness becomes more apparent [27,28]. It is possible that in the early stage of DCM, the inferior part of the right precentral gyrus has a spatial reorganization of the neurite structures in the subcortical white matter to compensate for the function loss caused by pathological changes in the gray matter in DCM patients. In fact, studies have proposed that primary sensory and motor regions, such as the pre- and postcentral gyri, have the potential to adaptively respond to environmental factors through plasticity mechanisms [29].

While the gray and white matter alterations described in sensorimotor and pain related regions were consistent with the hypotheses of the current study, the left pars triangularis, the right superior temporal gyrus, and the right caudal middle frontal gyrus also demonstrated cortical alterations. Since DCM is primarily a sensorimotor disorder, alterations within temporal and inferior frontal cortices may appear distinct from the sensorimotor and nociceptive networks that DCM is expected to affect. However, it is likely that neurological deterioration occurs in a relatively rapid fashion when adaptive changes in sensorimotor and pain-related regions reach their limits. Another possible interpretation is related to the aging process. It has been suggested that dorsal frontal and temporal cortices, as well as regions of the association cortex, may undergo age-related cortical modifications due to genetic effects [30,31,32]. The fact that the temporal lobe and the inferior frontal gyrus showed a degree of thinning related to DCM severity, but no relationship between ICVF values and cortical thickness, may be due to age-related cortical modifications in the patient cohort. Thus, NODDI metrics of DCM patients may provide biomarkers more specific and related to DCM severity rather than cortical thickness.

### 4.2. Clinical Implications

The current study suggests that DCM is a complex disease, and its pathogenesis is not simply confined to the spinal cord. A growing body of literature suggests that complex microstructural changes occur within the brain, likely to compensate for spinal cord damage in attempt to preserve neurological function. ASCC patients are commonly encountered in daily clinical practice, and subclinical microstructural spinal cord injury occurs in this patient population [14]. A complete understanding of how supraspinal adaptation influences the progression of symptoms in ASCC and DCM patients would be a major advancement for our field.

### 4.3. Limitations

There are several limitations in this study that should be noted. The current results provided additional evidence and insights into how the correlation between thinning cortices and underlying microstructures can serve as a potential biomarker reflecting the pathology and progression of DCM. A longitudinal study will be necessary to track how the microstructural connectivity changes in response to cortical atrophy. Additionally, although age was included as a covariate in the statistical analysis of the association between morphological measures and neurological status and pain severity, data from additional older HCs and/or additional younger DCM patients could help determine whether cortical alterations within the temporal lobe and the inferior frontal gyrus are associated with DCM, thus further strengthening the evidence in support of the correlation. Moreover, future studies on the cortical comparisons between HCs and ASCC patients would significantly benefit understanding of DCM progression.

## 5. Conclusions

In summary, we were able to demonstrate that ASCC patients and DCM patients experience alterations in cortical thickness that is reflective of underlying microstructural changes. These findings could be used to quantitatively evaluate cerebral alterations, providing imaging biomarkers that more reliably predict a patient’s clinical course, response to therapy, and long-term prognosis, thus enabling clinicians to better predict potential surgical and treatment outcomes for DCM patients.

## Figures and Tables

**Table 1:** Cohort Demographics for Diffusion Tensor Imaging Analysis.

Subject	Age (Mean ± SD) [Min, Max]	Subgroup	mJOA (Mean ± SD) [Min, Max]
**Patient Cohort ** **(*n* = 44) **	59.9 ± 11.6 [37, 81]	DCM (*n* = 37)	14.7 ± 2.3 [9, 17]
		Asymptomatic with spinal cord compression (*n* = 7)	18
**HCs** **(*n* = 17) **	41.0 ± 14.0 [25, 62]	N/A	18

DCM = degenerative cervical myelopathy; HCs = healthy controls; *n* = number; SD = standard deviation; N/A = not available; and mJOA = modified Japanese orthopedic association.

**Table 2:** Thinning Cortical Regions Showing Differences in ICVF Values between Study Patients and HCs.

Cortical Regions	Average Age Corrected ICVF		
	Study Patients	HCs	*p*-Value
Precentral Gyrus l	−0.0193	0.1735	<0.0001 **
Precentral Gyrus r	−0.0073	0.1259	<0.0001 **
Postcentral Gyrus l	−0.0196	0.1854	<0.0001 **
Caudal Anterior Cingulate r	−0.0134	0.1531	<0.0001 **
Rostral Anterior Cingulate r	−0.0165	0.1698	<0.0001 **
Supramarginal Gyrus l	−0.0142	0.1617	<0.0001 **
Superior Frontal Gyrus r	−0.0078	0.1203	<0.0001 **
Insular r	−0.0159	0.1774	<0.0001 **
Pars Opercularis l	−0.0209	0.1760	<0.0001 **
Pars Triangularis l	−0.0133	0.1519	<0.0001 **
Caudal Middle Frontal Gyrus r	−0.0139	0.1504	<0.0001 **
Superior Temporal Gyrus r	−0.0210	0.1773	<0.0001 **

r = right; l = left; ** = extremely significant; HCs = healthy controls; and ICVF = intra-cellular volume fraction.

**Table 3:** Thinning Cortical Regions Showing Correlation between ICVF Values and Cortical Thickness in both Study Patients and HCs.

Cortical Regions	Correlation with Age Corrected ICVF			
	Study Patients		HCs	
	*r*-Value	*p*-Value	*r*-Value	*p*-Value
Precentral Gyrus l	0.5715	<0.0001 **	0.1632	0.5314
Precentral Gyrus r	0.2111	0.1691	0.0080	0.9757
Postcentral Gyrus l	0.5195	0.0003 *	0.3932	0.1184
Caudal Anterior Cingulate r	0.4748	0.0011 *	0.3351	0.1886
Rostral Anterior Cingulate r	0.3927	0.0084 *	0.2652	0.3038
Supramarginal Gyrus l	0.3847	0.0099 *	0.4317	0.0836
Superior Frontal Gyrus r	0.3266	0.0305 *	0.4773	0.0527
Insular r	−0.0186	0.9059	0.0574	0.8267
Pars Opercularis l	0.4681	0.0014 *	0.2617	0.3102
Pars Triangularis l	0.2006	0.1922 *	0.1852	0.4767
Caudal Middle Frontal Gyrus r	0.1222	0.4296	0.0670	0.7897
Superior Temporal Gyrus r	0.1052	0.4969	0.2366	0.3605

r = right; l = left; * = significant ** = extremely significant; HCs = healthy controls; and ICVF = intra-cellular volume fraction.

## Data Availability

The data presented in this study are available on request from the corresponding author.
